# Downregulation of the Cd38-Cyclic ADP-Ribose Signaling in Cardiomyocytes by Intermittent Hypoxia via Pten Upregulation

**DOI:** 10.3390/ijms23158782

**Published:** 2022-08-07

**Authors:** Shin Takasawa, Mai Makino, Tomoko Uchiyama, Akiyo Yamauchi, Sumiyo Sakuramoto-Tsuchida, Asako Itaya-Hironaka, Yoshinori Takeda, Keito Asai, Ryogo Shobatake, Hiroyo Ota

**Affiliations:** 1 Department of Biochemistry, Nara Medical University, 840 Shijo-cho, Kashihara 634-8521, Nara, Japan; 2 Department of Diagnostic Pathology, Nara Medical University, 840 Shijo-cho, Kashihara 634-8522, Nara, Japan; 3 Department of Obstetrics and Gynecology, Nara Medical University, 840 Shijo-cho, Kashihara 634-8522, Nara, Japan; 4 Department of Neurology, Nara Medical University, 840 Shijo-cho, Kashihara 634-8521, Nara, Japan; 5 Department of Respiratory Medicine, Nara Medical University, 840 Shijo-cho, Kashihara 634-8522, Nara, Japan

**Keywords:** intermittent hypoxia, sleep apnea syndrome, Cd38, Ryr2, Fkbp12.6, Pten, cardiomyocytes

## Abstract

Sleep apnea syndrome (SAS) is characterized by recurrent episodes of oxygen desaturation and reoxygenation (intermittent hypoxia, IH), and it is a risk factor for cardiovascular disease (CVD) and insulin resistance/type 2 diabetes. However, the mechanisms linking IH stress and CVD remain elusive. We exposed rat H9c2 and mouse P19.CL6 cardiomyocytes to experimental IH or normoxia for 24 h to analyze the mRNA expression of the components of Cd38-cyclic ADP-ribose (cADPR) signaling. We found that the mRNA levels of cluster of differentiation 38 (*Cd38*), type 2 ryanodine receptor (*Ryr2*), and FK506-binding protein 12.6 (*Fkbp12.6*) in H9c2 and P19.CL6 cardiomyocytes were significantly decreased by IH, whereas the promoter activities of these genes were not decreased. By contrast, the expression of phosphatase and tensin homolog deleted from chromosome 10 (Pten) was upregulated in IH-treated cells. The small interfering RNA for Pten (siPten) and a non-specific control RNA were introduced into the H9c2 cells. The IH-induced downregulation of *Cd38*, *Ryr2*, and *Fkbp12.6* was abolished by the introduction of the siPten, but not by the control RNA. These results indicate that IH stress upregulated the Pten in cardiomyocytes, resulting in the decreased mRNA levels of *Cd38*, *Ryr2*, and *Fkbp12.6*, leading to the inhibition of cardiomyocyte functions in SAS patients.

## 1. Introduction

Sleep apnea syndrome (SAS) is a common disorder characterized by repetitive episodes of oxygen desaturation during sleep, as well as the development of daytime sleepiness and by the deterioration of patients’ quality of life [1,2]. SAS leads to intermittent hypoxia (IH) [3,4,5], hypercapnia, and subsequent reoxygenation; it also leads to the disruption of sleep architecture, such as sleep fragmentation. It is estimated that nearly 1 billion adults aged 30–69 worldwide may suffer from SAS [6]. SAS is associated with many systemic complications, such as obesity, type 2 diabetes [7,8], dyslipidemia [9], cardiovascular diseases (CVDs) (e.g., hypertension, coronary disease, heart failure, and stroke) [10,11,12], pulmonary hypertension [13], systemic hypertension [14], neurocognitive deficits [15,16], depression [17], and impaired memory [18].

Observational studies have indicated that SAS is associated with a high risk for serious hypertension [19]. IH-induced cardiomyocyte damage occurs due to the increases in intracellular reactive oxygen species during reoxygenation after hypoxia [20,21,22,23]. Moreover, IH may cause lipid peroxidation [24], protein oxidation, DNA damage [25], and attenuation of antioxidant enzyme capacity, reducing the number of cardiomyocytes and impairs their functions [26]. The prevalence of SAS in patients with heart failure ranges from 15% to 59%, and the mortality rate among patients with severe SAS is significantly higher [27,28,29,30,31]. In addition, cardiac function is impaired due to left ventricular hypertrophy in obese patients with severe SAS [32]. Hypertension, cardiac remodeling, and other complications of SAS have been studied using rodent models of IH [33]. The cluster of differentiation 38 (Cd38)-cyclic ADP-ribose (cADPR) signaling system is important for mammalian cell functioning [31,34], such as glucose-induced insulin secretion from pancreatic β-cells [34,35,36,37,38,39,40], muscarinic Ca^2+^ signaling in pancreatic acinar cells [41], depolarization-induced oxytocin secretion from neuro-pituitary cells [42], ATP-activated potassium currents in alveolar macrophages [43], electrolyte secretion from airway glands [44], aorta contraction induced by α-adrenoceptor stimulation [45], IH-induced renin synthesis in juxtaglomerular cells [46], and cardiac functions [47,48,49,50]. However, it was unclear how the Cd38–cADPR signal system was associated with cardiac dysfunction during IH.

Phosphatase and tensin homolog deleted from chromosome 10 (Pten), also known as MMAC1 (mutated in multiple advanced cancer 1) or TEP1 (TGFβ regulated and epithelial cell enriched phosphatase 1), was first identified as a tumor suppressor gene [51]. Pten acts as a dual-specificity phosphatase that phosphorylates lipids and proteins on serine, threonine, and tyrosine residues [52]. Downregulation of Pten expression or inhibition of its activity improves heart function, promotes cardiomyocytes proliferation, reduces cardiac fibrosis as well as dilation, and inhibits apoptosis following ischemic stress such as myocardial infarction [53]. Overexpression of Pten was reported to reduce Cd38 expression in airway smooth muscle cells [54]. As the Cd38–cADPR signal system plays an important role in cardiomyocyte functions [48,49,55,56], the relation between the expression of components of the Cd38–cADPR signal system (Cd38, Ryr2, and Fkbp12.6) and Pten is important in cardiomyocyte functioning in IH condition.

In this study, we used rat and mouse cardiomyocytes and an in vitro IH system to investigate the direct effect of IH, a hallmark of SAS, on the gene expression(s) of the Cd38–cADPR signal system in cardiomyocytes. An in vitro IH system is a controlled gas delivery system that regulates the flow of nitrogen and oxygen to generate IH. Significant decreases in the mRNA levels of *Cd38*, *type 2 ryanodine receptor* (*Ryr*)*2*, and *FK506-binding protein 12.6* (*Fkbp12.6*) were detected in the rat and mouse cardiomyocytes in response to IH treatment via the upregulation of Pten.

## 2. Results

### 2.1. The Gene Expression Levels of Cd38, Ryr2, and Fkbp12.6 in Cardiomyocytes Were Decreased by IH

**Figure 1:**
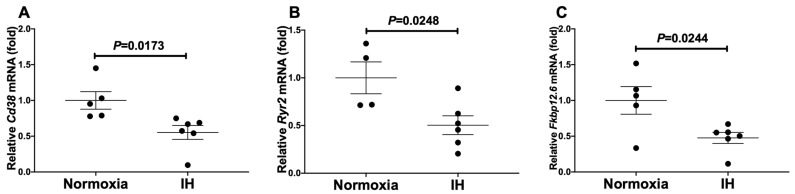
The mRNA levels of mouse (**A**) *Cd38*, (**B**) *Ryr2*, and (**C**) *Fkbp12.6* in mouse P19.CL6 cardiomyocytes. Cardiomyocytic-differentiated mouse P19.CL6 cells were treated with normoxia or IH for 24 h. The mRNA levels were measured by real-time RT-PCR and normalized by *rat insulinoma gene (Rig)/ribosomal protein S15 (RpS15)* as an internal standard. The mRNA level exposed to normoxia was set to 1.0. Data are expressed as the mean ± SE of the samples (*n* = 4 to 6). Statistical analyses were performed using Student’s *t*-test. IH significantly decreased the mRNA levels of *Cd38*, *Ryr2*, and *Fkbp12.6* in mouse P19.CL6 cells. In addition, correlation analyses revealed that the correlation coefficient(s) between *Cd38* vs. *Ryr2*, *Cd38* vs. *Fkbp12.6*, and *Ryr2* vs. *Fkbp12.6* were 0.618, 0.912, and 0.566, respectively, indicating that there are positive correlation(s).

**Figure 2:**
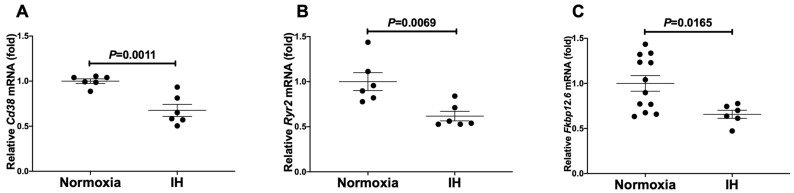
The mRNA levels of rat (**A**) *Cd38*, (**B**) *Ryr2*, and (**C**) *Fkbp12.6* in rat H9c2 cardiomyocytes. Rat H9c2 cells were treated with normoxia or IH for 24 h. The mRNA levels were measured by real-time RT-PCR and normalized by *Rig/RpS15* as an internal standard. The mRNA level exposed to normoxia was set to 1.0. Data are expressed as the mean ± SE of the samples (*n* = 4 to 6). Statistical analyses were performed using Student’s *t*-test. IH significantly decreased the mRNA levels of *Cd38*, *Ryr2*, and *Fkbp12.6* in rat H9c2 cells.

We exposed mouse P19.CL6 and rat H9c2 cardiomyocytes to normoxia or IH for 24 h. Using real-time reverse transcriptase-polymerase chain reaction (RT-PCR), we measured the mRNA levels of *Cd38* (which encodes ADP-ribosyl cyclase/cyclic ADP-ribose [cADPR] hydrolase, EC 3.2.2.6), *Ryr2*, and *Fkbp12.6* (a cADPR receptor [*Fkbp1b*]) in mouse P19.CL6 and rat H9c2 cells. As shown in Figure 1 and Figure 2, IH significantly decreased the mRNA levels of *Cd38*, *Ryr2*, and *Fkbp12.6* in mouse P19.CL6 and rat H9c2 cardiomyocytes, respectively.

**Figure 3:**
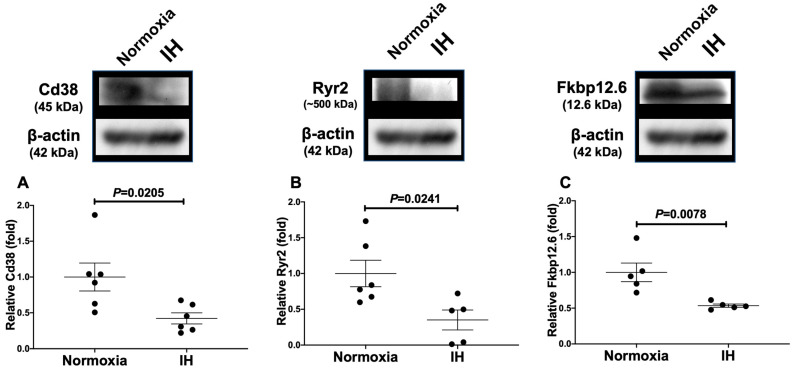
Relative protein expression levels of (**A**) Cd38, (**B**) Ryr2, and (**C**) Fkbp12.6 in rat H9c2 cardiomyocytes subjected to IH. The Cd38, Ryr2, and Fkbp12.6 band densities were quantified through image analysis and then normalized to β-actin, as measured in the same blot. Each bar represents the mean of six independent measurements. The relative expression levels of the Cd38, Ryr2, and Fkbp12.6 are arbitrarily presented. The protein level exposed to normoxia was set to 1.0. The results are expressed as the mean ± SE in arbitrary units. A representative immunoblot with the apparent molecular weight is shown in the upper panel.

Furthermore, we measured the Cd38, Ryr2, and Fkbp12.6 protein levels in the H9c2 cells using Western blot analyses, and the results showed that the protein levels were significantly decreased by IH (*p* = 0.0205, *p* = 0.0241, and *p* = 0.0078, respectively) (Figure 3).

### 2.2. The Promoter Activities of Cd38, Ryr2, and Fkbp12.6 Were Not Decreased by IH

**Figure 4:**
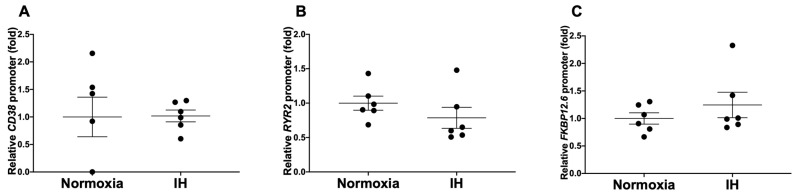
Luciferase assays of the promoter activities of (**A**) *CD38*, (**B**) *RYR2*, and (**C**) *FKBP12.6* in H9c2 cardiomyocytes. Reporter plasmids, prepared by inserting the promoter fragments of human *CD38* (−3187~+269), human *RYR2* (−1250~+10), and human *FKBP12.6* (−1696~+109) upstream of a firefly luciferase reporter gene in pGL4.17 vector, were transfected into rat H9c2 cells. After the cells were exposed either to IH or to normoxia for 24 h, the cells were lysed and the promoter activities of *CD38*, *RYR2*, and *FKBP12.6* were measured. The promoter activity was normalized for variations in transfection efficiency, with β-galactosidase activity as an internal standard and the promoter activity of cells exposed to normoxia was set to 1.0. Data are presented as the mean ± SE of the samples (*n* = 5 to 6) and were analyzed using Student’s *t*-test.

To determine whether the IH-induced decreases in the *Cd38*, *Ryr2*, and *Fkbp12.6* mRNA levels were caused by the inactivation of transcription, we fused a 3456 bp fragment containing 3187 bp of the human *CD38* promoter, a 1260 bp fragment containing 1250 bp of the human *RYR2* promoter, and a 1805 bp fragment containing 1696 bp of the human *FKBP12.6* promoter to the luciferase gene of pGL4.17. The reporter constructs were transfected into H9c2 cardiomyocytes. After IH stimulation, the promoter activities of *CD38*, *RYR2*, and *FKBP12.6* were not decreased by IH in H9c2 cardiomyocytes (*p* = 0.9630, *p* = 0.2735, and *p* = 0.3563, respectively) (Figure 4), suggesting that the gene expression levels of *Cd38*, *Ryr2*, and *Fkbp12.6* in response to IH were not regulated by transcription.

### 2.3. The Pten Level Was Significantly Increased by IH

**Figure 5:**
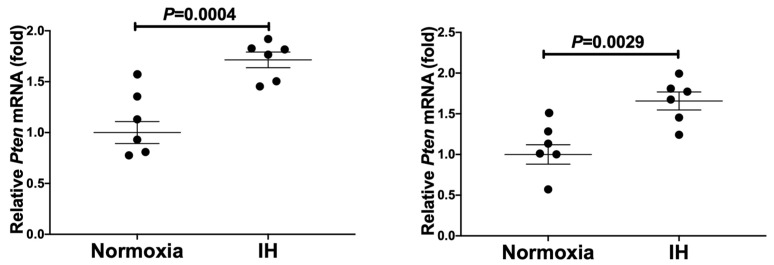
The mRNA levels of *Pten* in rat H9c2 (**left**) and mouse P19.CL6 (**right**) cardiomyocytes. Rat H9c2 and cardiomyocytic-differentiated mouse P19.CL6 cells were treated with normoxia or IH for 24 h. The mRNA levels were measured by real-time RT-PCR and normalized by *Rig/RpS15* as an internal standard. The mRNA level exposed to normoxia was set to 1.0. Data are expressed as mean ± SE of the samples (*n* = 4 to 6). Statistical analyses were performed using Student’s *t*-test. IH significantly increased the mRNA levels of *Pten* in rat H9c2 and mouse P19.CL6 cardiomyocytes. Correlation analysis in P19.CL6 cells revealed that the correlation coefficient(s) between *Cd38* vs. *Pten* and *Fkbp12.6* vs. *Pten* were −0.221 and −0.362, respectively, indicating that there are negative correlation(s).

Wu et al. recently reported that overexpression of Pten suppresses the expression of CD38 in airway smooth muscle cells [51]. Therefore, it is possible that the IH-induced downregulation of the Cd38, Ryr2, and Fkbp12.6 is possibly caused by the upregulation of Pten. We exposed rat H9c2 and mouse P19.CL6 cardiomyocytes to normoxia or IH and determined the *Pten* mRNA levels using real-time RT-PCR. We found that the *Pten* mRNA levels in both H9c2 and P19.CL6 cells were significantly increased in response to IH exposure (Figure 5).

**Figure 6:**
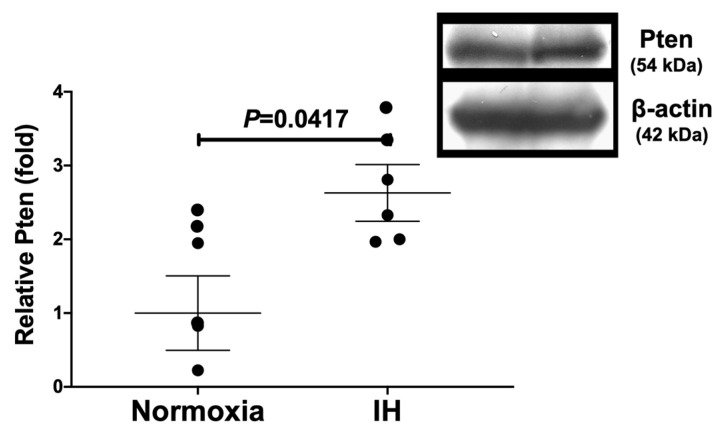
Relative protein expression of Pten in rat H9c2 myocytes subjected to IH. The Pten band densities were quantified through image analysis and then normalized to β-actin, as measured in the same blot. The protein level exposed to normoxia was set to 1.0. Each bar represents the mean value of six independent experiments (*n* = 6). The relative expression of the Pten is arbitrarily presented. The results are expressed as mean ± SE in arbitrary units. A representative immunoblot with the apparent molecular weight is also shown in the right panel.

We further measured the Pten protein levels in H9c2 cells using Western blot analysis, and we confirmed that the level of Pten was significantly increased by IH (Figure 6: *p* = 0.0417).

### 2.4. Down-Regulation of Pten Attenuated the Decreases in Cd38, Ryr2, and Fkbp12.6 in H9c2 Cells Treated with Small Interfering RNA (siRNA) for Pten

**Figure 7:**
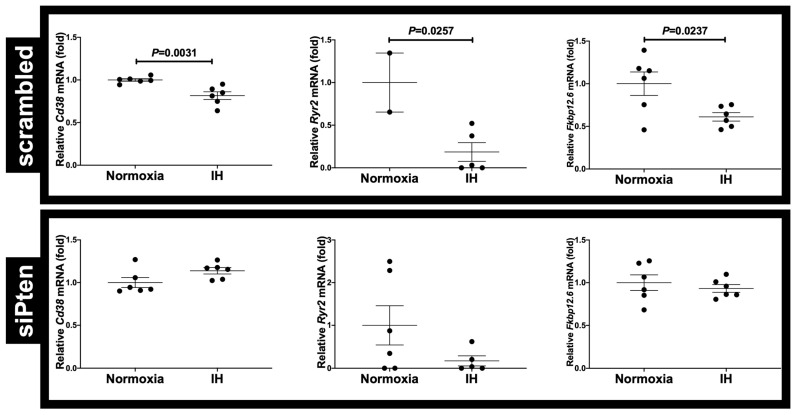
Effects of *siPten* on the IH-induced gene expression of *Cd38*, *Ryr2*, and *Fkbp12.6*. *SiPten* and scrambled RNA (control) were transfected into H9c2 cardiomyocytes, which in turn were subjected to IH or normoxia for 24 h. The mRNA levels of the *Cd38*, *Ryr2*, and *Fkbp12.6* were measured via real-time RT-PCR, with *Rig/RpS15* as the endogenous control. The mRNA level exposed to normoxia was set to 1.0. Data are expressed as the mean ± SE of six independent experiments (*n* = 6). Student’s *t*-test was used in statistical analyses.

The mechanism of Cd38, Ryr2, and Fkbp12.6 expression in cardiomyocytes was investigated by knocking down the *Pten* gene by means of RNA interference in rat H9c2 cells. The expression levels of *Cd38*, *Ryr2*, and *Fkbp12.6* were significantly decreased by IH in the presence of scrambled RNA. By contrast, the introduction of *siPten* inhibited the IH-induced decreases in the mRNA levels of *Cd38*, *Ryr2*, and *Fkbp12.6* in H9c2 cells (Figure 7). These results indicated that the observed decreases in the *Cd38*, *Ryr2*, and *Fkbp12.6* levels in response to IH were caused by *Pten* expression.

### 2.5. 3-Deaza-cADPR Attenuated the IH-Induced Decreases in the Cd38, Ryr2, and Fkbp12.6 Levels

**Figure 8:**
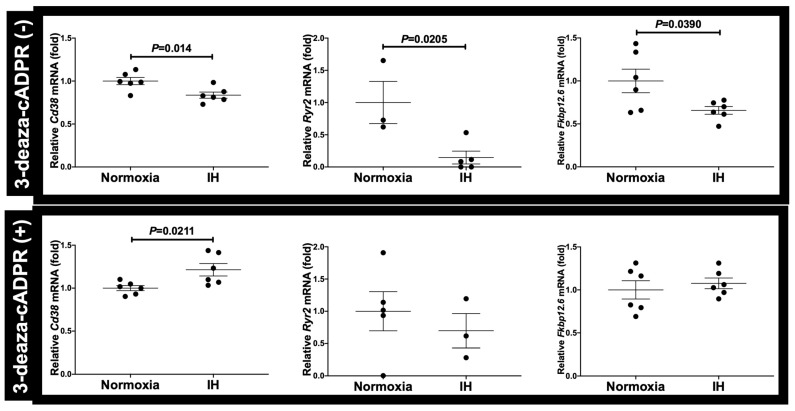
The mRNA levels of *Cd38*, *Ryr2*, and *Fkbp12.6* in the presence or absence of 3-deaza-cADPR. No differences in *Ryr2* and *Fkbp12.6* mRNA levels were observed under normoxia and under IH in the presence of 3-deaza-cADPR (0.6979-fold decrease in 3-deaza-cADPR (+) (*p* = 0.5260) and 1.076-fold increase in 3-deaza-cADPR (+) (*p* = 0.5501), respectively). By contrast, the *Cd38* mRNA level under IH increased by 1.214-fold relative to that under normoxia in the presence of 3-deaza-cADPR (*p* = 0.0211). Although the mRNA levels of *Cd38*, *Ryr2*, and *Fkbp12.6* decreased in response to IH in the absence of 3-deaza-cADPR (3-deaza-cADPR (−) controls), this trend was not observed following the addition of the 3-deaza-cADPR (3-deaza-cADPR (+)). The mRNA level exposed to normoxia was set to 1.0. Data are expressed as the mean ± SE of six independent experiments (*n* = 6). Student’s *t*-test was employed in statistical analyses.

CD38 was originally found to be a surface antigen/marker of B lymphocytes, monocytes, and natural killer cells [57]. In 1993, three research groups independently found cADPR synthesizing activity in Cd38 [58,59,60]. cADPR binds to Fkbp12.6 to dissociate Fkbp12.6 from the Ryr2 complex and induces Ca^2+^ release from the Ryr2 intracellular Ca^2+^ channel [38,61]. To confirm the correlation between the expression levels of Cd38, Ryr2, and Fkbp12.6 and the Cd38–cADPR-mediated signaling pathway, we added 3-deaza-cADPR, a cell-permeable cADPR agonist [62,63], into H9c2 cell culture medium, and then we subjected the cells to normoxia or IH for 24 h. Following IH stimulation, the mRNA levels of *Cd38*, *Ryr2*, and *Fkbp12.6* were measured. The results showed that the IH-induced decreases in the mRNA levels of *Cd38*, *Ryr2*, and *Fkbp12.6* were attenuated in the presence of 3-deaza-cADPR (Figure 8), indicating that the observed reduction in the mRNA levels in response to IH was induced by the inhibition of the Cd38–cADPR-mediated signaling pathway.

## 3. Discussion

In this study, we demonstrated that IH exposure induced the reduction in *Cd38*, *Ryr2*, and *Fkbp12.6* mRNA levels in cardiomyocytes. We further studied the mechanisms by which IH downregulates the mRNA levels of *Cd38*, *Ryr2,* and *Fkbp12.6*, and we speculated that Pten-mediated downregulation is involved in the process. We then knocked down Pten, and the IH-induced downregulation of Cd38, Ryr2, and Fkbp12.6 was not observed in *siPten*-transfected cardiomyocytes, suggesting that the dysfunction of the Cd38–cADPR signal system in cardiomyocytes is induced by IH via the overexpression of Pten. To verify this possibility, we added 3-deaza-cADPR, a cell-permeable cADPR agonist, into the cardiomyocyte culture medium and found that the IH-induced decreases in Cd38, Ryr2, and Fkbp12.6 were attenuated, indicating that IH indeed reduces Cd38 expression, Cd38 activity (cADPR synthesizing ADP-ribosyl cyclase activity), and cADPR concentration in cardiomyocytes. The reduced cADPR concentration in turn decreased the recipients of cADPR, Ryr2, and Fkbp12.6 in cardiomyocytes.

SAS patients frequently suffer from CVDs such as hypertension, coronary disease, and heart failure [10,11,12]. The prevalence of SAS in patients with heart failure ranges from 15% to 59%, and the mortality rate among patients with severe SAS is significantly high [27,28,29,30]. We recently reported that IH increases renin expression in juxtaglomerular cells [46], as well as the expression of dopamine β-hydroxylase and phenylethanolamine *N*-methyltransferase in catecholamine-synthesizing neuroblastoma cells [64], causing SAS patients to become hypertensive. However, how cardiac dysfunction develops in SAS patients remains elusive.

cADPR was first discovered by Dr. Lee and his collaborators in 1987 in sea urchin eggs as an intracellular signaling molecule that acts inositol 1,4,5-trisphosphate (IP_3_)-insensitive intracellular Ca^2+^ channel that releases Ca^2+^ [65]. Cd38 was eventually found to be a major enzyme for cADPR synthesis from NAD^+^ in vertebrate cells [58,59,60,66]. cADPR induces Ca^2+^ mobilization from IP_3_-insensitive intracellular Ca^2+^ stores via the Ryr2 Ca^2+^ channels [34,47,67]. Dissociation of Fkbp12.6 from Ryr2 is required to release Ca^2+^ from intracellular stores, and it is regulated by cADPR [50,61]. The significance of the Cd38–cADPR signal system has been demonstrated in a number of vertebrate cell functions, including glucose-induced insulin secretion from pancreatic β-cells [34,35,37,38,40,68], acetylcholine-induced Ca^2+^ oscillation in pancreatic exocrine cells [41], submandibular gland acinar cell activation [44], ATP-induced K^+^ currents in alveolar macrophages [43], sympathetic neuron activation [69], oxytocin secretion from the pituitary gland [42], renin synthesis and secretion in juxtaglomerular cells [46], and cardiac functions [48,49,55,56].

The Cd38–cADPR signaling pathway has been reported to antagonize cardiomyocyte differentiation of mouse embryonic stem cells [70]. Meanwhile, cADPR activates Ryr2 [47] in cardiomyocytes, Cd38 is expressed in heart [71], isoproterenol increases ADP-ribosyl cyclase activity of Cd38 in ventricular muscle [72], cADPR levels are decreased in rat myocardial ischemia [73], male Cd38 knockout mice have shown cardiac hypertrophy [49], Ryr2 knockout mice have died on embryonic day 10 with morphological abnormalities in their heart tube [74], altered stoichiometry of FKBP12.6 relative to that of RYR2 has been reported to a cause abnormal Ca^2+^ leak through RYR2 in heart failure [48], and Fkbp12.6 knockout male mice have shown cardiac hypertrophy [55], indicating that the Cd38–cADPR signaling pathway is indispensable in cardiomyocyte functioning.

Pten acts as a phosphatase that dephosphorylates phosphatidylinositol 3,4,5-triphosphate (PIP_3_). Pten specifically catalyzes the dephosphorylation of the 3′ phosphate of the inositol ring in PIP_3_, producing a biphosphate product (phosphatidylinositol 4,5-bisphosphate). This dephosphorylation is important because it results in the inhibition of the Akt signaling pathway, which plays a key role in multiple signal transduction pathways [75]. Pten is involved in the regulation of cellular processes, including cell survival, proliferation, and migration, and it participates in diverse physiological and pathological processes [75]. Overexpression of Pten suppresses the expression of Cd38 in airway smooth muscle cells [54]. PTEN expression is increased by RYR2 knockdown, whereas increased RYR2 expression inhibits PTEN expression in pancreatic cancer cells [76]. In adult mice, cardiac-specific Pten knockout preserves heart function, decreases scar size, and promotes cardiomyocyte proliferation after myocardial infarction stress [77]. Recently, Ashikawa et al. have reported that intraperitoneal injection of a Pten inhibitor, bisperoxovanadium-pic, ameliorated left ventricular inflammation, fibrosis, and diastolic dysfunction in DS/Obese (DahlS.Z-lepr^fa^/Lepr^fa^) rats [78]. In the present study, IH reduced the expression of the components of the Cd38–cADPR signal system in cardiomyocytes via Pten overexpression; therefore, Pten inhibitors can preserve cardiac cell functions and may serve as new drugs for cardiomyocyte functioning.

This study demonstrated that the gene expression levels of *Cd38*, *Ryr2*, and *Fkbp12.6* decreased via the upregulation of Pten in IH-treated cardiomyocytes. It is suggested that, in SAS patients, downregulation of *Cd38*, *Ryr2*, and *Fkbp12.6* may decrease the function of the Cd38–cADPR signaling in cardiomyocytes, leading to the failure of cardiac functions.

## 4. Materials and Methods

### 4.1. Cell Culture

Rat H9c2 cardiomyocytes were purchased from the American Type Culture Collection (Manassas, VA, USA). The cells were maintained in Dulbecco’s Modified Eagle Medium (DMEM) (FUJIFILM Wako Pure Chemical Corporation, Osaka, Japan) containing 10% (v/v) fetal calf serum (FCS), 100 units/mL penicillin G (FUJIFILM Wako), and 100 µg/mL streptomycin (FUJIFILM Wako). Mouse embryonic carcinoma P19.CL6 cells were purchased from RIKEN BioResource Research Center Cell Bank (RCB, Tsukuba, Japan). The cells were grown in Minimum Essential Medium Eagle, Alpha Modification (MEMα) medium (FUJIFILM Wako) containing 10% (v/v) FCS, 100 units/mL penicillin G, and 100 µg/mL streptomycin. For differentiation experiments, 3.7 × 10^5^ cells/0.5 mL were seeded in a 24-well cell culture plate with MEMα medium containing 1% DMSO to induce cardiomyogenesis, as previously described [79]. The cells were kept at 37 °C, 5% CO_2_, and 95% humidity, and the medium was changed every day. The cells were exposed to either normoxia (21% O_2_, 5% CO_2_, and balanced N_2_) or intermittent hypoxia (IH: 70 cycles of 5 min sustained hypoxia (1% O_2_, 5% CO_2_, and balanced N_2_) and 10 min normoxia) using a custom-designed, computer-controlled incubation chamber attached to an external O_2_-CO_2_-N_2_ computer-driven controller (O_2_ programmable control, 9200EX, Wakenbtech Co., Ltd., Kyoto, Japan), as previously described [4,40,46,64,80,81]. These conditions are similar to the conditions reported in patients with severe degrees of SAS; in severe SAS cases, patients are repeatedly exposed to severe hypoxemia followed by mild hypoxemia or normoxia (i.e., IH). We have previously reported that the magnitude of IH expressed by SpO_2_ fluctuates between 75–98% and 50–80% in SAS [3,4,5], which was nearly equivalent to the medium condition in the present study.

### 4.2. Real-Time RT-PCR

Total RNA was isolated from H9c2 and P19.CL6 cells using an RNeasy Plus Cell Mini Kit (Qiagen, Hilden, Germany), and cDNA was synthesized from total RNA as a template using a High Capacity cDNA Reverse Transcription Kit (Applied Biosystems, Foster City, CA, USA), as previously described [40,46,64,80,81]. Real-time PCR was performed using an SYBR^®^ Fast qPCR Kit (KAPA Biosystems, Boston, MA, USA) and a Thermal Cycler Dice Real Time System (Takara Bio, Kusatsu, Japan). All the PCR primers were synthesized by Nihon Gene Research Laboratories, Inc. (NGRL; Sendai, Japan); the primer sequences for each primer set are shown in Table 1. PCR was performed with an initial step of 3 min at 95 °C, followed by 40 cycles of 3 s at 95 °C and 20 s at 60 °C for *Rig/RpS15*, *Cd38, Ryr2, Fkbp12.6,* and *Pten*. The mRNA expression levels were normalized to the mRNA level of *Rig/RpS15*, as previously described [40,46,64,80,81].

### 4.3. Immunoblot Analysis

H9c2 cardiomyocyte extract (5 × 10^5^ cells) was subjected to immunoblot analysis as previously described [46,64], using an anti-Cd38 polyclonal antibody (Santa Cruz Biotechnology, Santa Cruz, CA, USA) raised against a peptide fragment of mouse Cd38 (residues 279–301 in [46]), anti-Ryr2 polyclonal antibody (PeproTech, Canbury, NJ, USA) [67], anti-Fkbp12.6 monoclonal antibody (Santa Cruz Biotechnology) raised against the amino acids 38–108 of human/rat/mouse FKBP12.6, anti-Pten monoclonal antibody raised against the full-length human PTEN protein (Abcam, Cambridge, UK), and anti-β-actin monoclonal antibody (Sigma, St. Louis, MO, USA) raised against Ac-Asp-Asp-Asp-Ile-Ala-Ala-Leu-Val-Ile-Asp-Asn-Gly-Ser-Gly-Lys. A SNAP id^®^ 2.0 Protein Detection System (Merck Millipore, Burlington, MA, USA) was used for the analysis. The band intensities were analyzed using ImageJ software (National Institute of Health, Bethesda, MD, USA), as previously described [35,46,64,82,83].

### 4.4. Construction of Reporter Plasmid and Luciferase Assay

Reporter plasmids were prepared by inserting the promoter fragments of human *CD38* (−3,187~+269), *RYR2* (−1,250~+10), and *FKBP12.6* (−1,696~+109) upstream of a firefly luciferase reporter gene in the pGL4.17[*luc2*/Neo] vector (Promega, Madison, WI, USA), respectively. The reporter plasmids were transfected into rat H9c2 cardiomyocytes using Lipofectamine^®^ 3000 (Invitrogen, Waltham, MA, USA), as previously described [40,46,64,80,81]. The cells were exposed to either 64 cycles/24 h of IH, mimicking cardiomyocytes of SAS patients, or to normoxia for 24 h. After the cells were exposed to IH, they were lysed, and promoter activities were measured. The cells were harvested, and cell extracts were prepared in an Extraction Buffer (0.1 M potassium phosphate, pH 7.8/0.2% Triton X-100; Life Technologies, Carlsbad, CA, USA). In monitoring transfection efficiency, pCMV•SPORT-βgal plasmid (Life Technologies) was co-transfected in all experiments at a 1:10 dilution. Luciferase activity was measured using a PicaGene Luciferase Assay System (Toyo-ink, Tokyo, Japan) and was normalized by the β-galactosidase activity as described previously [40,46,64,80,81,84].

### 4.5. RNA Interference

The siRNA directed against rat *Pten* was synthesized by NGRL. The sense sequence of the siRNA for the rat *Pten* was 5′-GGAACAAUAUUGAUGAUGUtt-3′ (corresponding to the residues 140–158 of NM_031606.1). Silencer^®^ Select scrambled siRNA was purchased from Ambion and was used as a control. The transfection of the siRNA into H9c2 cells was performed using Lipofectamine^®^ RNAiMAX Transfection Reagent (Thermo Fisher Scientific, Waltham, MA, USA). The cells were each transfected with 5 pmol of each siRNA in a 24-well culture dish, as previously described [40,46,80,81,84].

### 4.6. Addition of 3-Deaza-cADPR

H9c2 cells were adjusted at 2 × 10^5^ cells/mL and the 0.5 mL cell suspension was seeded into each well of a 24-well plate. After incubation at 37 °C overnight, the medium was replaced with fresh medium with or without 3-deaza-cADPR (Sigma; finally adjusted to 10 nM). The cells were further incubated at 37 °C in an IH or normoxia condition for 24 h. Cellular RNA preparation and real-time RT-PCR were performed as described in Section 4.2.

### 4.7. Data Analysis

The results are expressed as mean ± SE. Statistical significance was determined using Student’s *t*-test performed in GraphPad Prism software (GraphPad Software, La Jolla, CA, USA).

## Figures and Tables

**Table 1:** PCR primers for real-time RT-PCR.

Target mRNA	Primer Sequence (Position)
Rat	
*Cd38*	5′-GAAAGGGAAGCCTACCACGAA-3′ (NM_013127.1: 166–186)
	5′-GCCGGAGGATTTGAGTATAGATCA-3′ (NM_013127.1: 219–242)
*Fkbp12.6*	5′-GGAAGGACATTCCCTAAGAAG-3′ (NM_022675.2: 174–194)
	5′-GTAGCTCCATATGCCACATCA-3′ (NM_022675.2: 374–394)
*Pten*	5′-AGACCATAACCCACCACAGC-3′ (NM_031606.1: 273–292)
	5′-TTACACCAGTCCGTCCTTTCC-3′ (NM_031606.1: 380–400)
*Rig/RpS15*	5′-ACGGCAAGACCTTCAACCAG-3′ (NM_017151.2: 314–333)
	5′-ATGGAGAACTCGCCCAGGTAG-3′ (NM_017151.2: 363–383)
*Ryr2*	5′-CTGAACTATTTTGCTCGCAA-3′ (NM_032078.3: 13802–13821)
	5′-TTCAGGCAGTAGTATCCGAT-3′ (NM_032078.3: 14093–14112)
Mouse	
*Cd38*	5′-ACAGACCTGGCTGCCGCCTCTCTAG-3′ (NM_007646.5: 102–126)
	5′-GGGGCGTAGTCTTCTCTTGTGATGT-3′ (NM_007646.5: 378–402)
*Fkbp12.6*	5′-GGAAGGACATTCCCTAAGAAG-3′ (NM_016863.4: 175–195)
	5′-GTAGCTCCATATGCCACATCA-3′ (NM_016863.4: 375–395)
*Pten*	5’-AGACCATAACCCACCACAGC-3’ (NM_008960.2: 1141–1160)
	5’-TTACACCAGTCCGTCCTTTCC-3’ (NM_008960.2: 1248–1268)
*Rig/RpS15*	5′-ACGGCAAGACCTTCAACCAG-3′ (NM_009091.2: 343–362)
	5′-ATGGAGAACTCGCCCAGGTAG-3′ (NM_009091.2: 392–412)
*Ryr2*	5′-gacagtcgagcgtgtcctgggtata-3′ (NM_023868.2: 11134–11158)
	5′-tgcttagagagtagtttgtgccaca-3′ (NM_023868.2: 11253–11277)

## Data Availability

The data are available on request from the authors.

## Abbreviations

cADPR	Cyclic ADP-ribose
CD38	Cluster of differentiation 38
CVD	Cardiovascular disease
DMEM	Dulbecco’s Modified Eagle Medium
FCS	Fetal calf serum
FKBP12.6	FK506-binding protein 12.6
IH	Intermittent hypoxia
MEMα	Minimum Essential Medium Eagle, Alpha Modification
RpS15	Ribosomal protein S15
Pten	Phosphatase and tensin homolog deleted from chromosome 10
Rig/RpS15	Rat insulinoma gene/Ribosomal protein S15
RT-PCR	Reverse transcriptase-polymerase chain reaction
SAS	Sleep apnea syndrome
siRNA	Small interfering RNA
RyR	Ryanodine receptor
